# Are current case-finding methods under-diagnosing tuberculosis among women in Myanmar? An analysis of operational data from Yangon and the nationwide prevalence survey

**DOI:** 10.1186/s12879-016-1429-y

**Published:** 2016-03-03

**Authors:** MS. Khan, TM. Khine, C. Hutchison, RJ. Coker, KM. Hane, AL. Innes, S. Aung

**Affiliations:** Communicable Diseases Policy Research Group, London School of Hygiene and Tropical Medicine, Satharanauskwisit Building, 420/1 Rajwithi Road, Bangkok, 10400 Thailand; Saw Swee Hock School of Public Health, National University of Singapore, Singapore, Singapore; National Tuberculosis Programme, Yangon, Myanmar; Faculty of Public Health, Mahidol University, Bangkok, Thailand; Family Health International Myanmar Office, Yangon, Myanmar; Family Health International Asia Pacific Regional Office, Bangkok, Thailand

**Keywords:** Tuberculosis, Gender, Myanmar

## Abstract

**Background:**

Although there is a large increase in investment for tuberculosis control in Myanmar, there are few operational analyses to inform policies. Only 34 % of nationally reported cases are from women. In this study, we investigate sex differences in tuberculosis diagnoses in Myanmar in order to identify potential health systems barriers that may be driving lower tuberculosis case finding among women.

**Methods:**

From October 2014 to March 2015, we systematically collected data on all new adult smear positive tuberculosis cases in ten township health centres across Yangon, the largest city in Myanmar, to produce an electronic tuberculosis database. We conducted a descriptive cross-sectional analysis of sex differences in tuberculosis diagnoses at the township health centres. We also analysed national prevalence survey data to calculate additional case finding in men and women by using sputum culture when smear microscopy was negative, and estimated the sex-specific impact of using a more sensitive diagnostic tool at township health centres.

**Results:**

Overall, only 514 (30 %) out of 1371 new smear positive tuberculosis patients diagnosed at the township health centres were female. The proportion of female patients varied by township (from 21 % to 37 %, *p* = 0.0172), month of diagnosis (37 % in February 2015 and 23 % in March 2015 *p* = 0.0004) and age group (26 % in 25–64 years and 49 % in 18–25 years, *p* < 0.0001). Smear microscopy grading of sputum specimens was not substantially different between sexes. The prevalence survey analysis indicated that the use of a more sensitive diagnostic tool could result in the proportion of females diagnosed at township health centres increasing to 36 % from 30 %.

**Conclusions:**

Our study, which is the first to systematically compile and analyse routine operational data from tuberculosis diagnostic centres in Myanmar, found that substantially fewer women than men were diagnosed in all study townships. The sex ratio of newly diagnosed cases varied by age group, month of diagnosis and township of diagnosis. Low sensitivity of tuberculosis diagnosis may lead to a potential under-diagnosis of tuberculosis among women.

## Background

After decades of isolation from the rest of the world, international development aid is pouring into Myanmar and information about the country’s health and development indicators is becoming accessible [1, 2]. Estimates of the burden of infectious diseases are concerning. The 2010 National Tuberculosis (TB) prevalence survey found that the bacteriologically confirmed tuberculosis prevalence rate was 612.8 (502.2–747.6) per 100 000 population (aged 15 years and above), which is among the highest in Asia [3]. Approximately 180,000 new tuberculosis cases and 9,000 new multi-drug resistant tuberculosis (MDR-TB) cases occur each year [4, 5]. Myanmar is also experiencing one of the most severe HIV/AIDS epidemics in Asia and there are approximately 20,000 new cases of TB/HIV co-infection every year [4].

The World Health Organization (WHO) global strategy to control tuberculosis, referred to as DOTS, is based largely on passive case detection by means of smear microscopy on sputum specimens obtained from symptomatic individuals presenting at DOTS affiliated health centres [6]. This case finding approach is used in the majority of resource-constrained countries, including Myanmar, because active case finding (outside of health centres), and use of more sensitive diagnostic tools, is too expensive to implement on a large scale. It is widely recognised, however, that tuberculosis cases are missed or diagnosed late when patients do not seek health care proactively or seek care at health facilities that do not report tuberculosis diagnoses through the DOTS system, such as most private clinics [7]. tuberculosis cases also go undiagnosed because of the low sensitivity of smear microscopy in detecting bacteria in sputum; a systematic review indicates that sensitivity of smear microscopy ranges from 0.32 to 0.94 [8]. There is an urgent need to understand better factors that increase the risk of delayed or missed tuberculosis diagnoses, especially in settings where substantial financial resources are being invested for tuberculosis control, and accordingly identify programmatic changes to address the barriers.

A large population group that may be selectively under-diagnosed with tuberculosis is women [9, 10]. Globally, there are more tuberculosis cases notified in men than women through the DOTS system [11]. There are four broad explanations for the observed sex differences in global tuberculosis notifications, with ongoing debate and inconclusive evidence about which factors are driving the sex difference [12, 13]:

1. Variations in levels of exposure to tuberculosis [10, 14, 15];
2. Differences in susceptibility to developing disease post exposure due to immunological and physiological factors [16–20];
3. Discrepancies in access to (DOTS) health centres for diagnosis and treatment [21–26];
4. Differences in the probability of being correctly diagnosed at (DOTS) health centres (women with tuberculosis have different symptoms to men, are referred for sputum testing less frequently or are less likely to be diagnosed by smear-microscopy because they have paucibacillary disease or give poor quality specimens) [27, 28].

The first two explanations point towards a potentially true difference in the incidence of disease between men and women, whereas the second two support the notion that the observed sex difference is likely due to more missed diagnoses of tuberculosis in women.

In South East Asia, only 40 % of tuberculosis diagnoses are from women [11]. Good quality data from Myanmar is limited owing to a lack of infrastructure (computers, regular electricity supply) and human resources for routine standardised data collection and management. The most recent estimates, from 2012, indicate that only 34 % of tuberculosis diagnoses in patients aged over 15 are from women [11]. While operational data from tuberculosis diagnostic centres has never been collated and analysed to identify groups that may be under-diagnosed, a countrywide prevalence survey was conducted in 2009–2010 [3]. The prevalence survey was administered through door-to-door screening for symptoms of tuberculosis, and sputum specimens from symptomatic patients were tested using both smear-microscopy and culture (which is more sensitive in diagnosing tuberculosis). Analysis of sex differences in operational data from tuberculosis diagnostic centres, and comparing this with sex differences identified in the prevalence survey, may provide important insights into programmatic/health system barriers (some of which could targeted for improvement) driving lower tuberculosis case finding among women in Myanmar.

In this study, we systematically collated operational data on new tuberculosis diagnosis (in Yangon, Myanmar) into a single electronic database, and investigated sex differences in tuberculosis notifications by comparing data from tuberculosis diagnostic centres and the prevalence survey.

## Methods

### Study setting

Yangon Division in Myanmar, the country’s main urban centre, has a population of 7.4 million people with a female to male ratio of 1.08 [29]. Yangon is divided into 44 townships each with its own township health centre, most of which have a designated tuberculosis diagnostic and treatment unit. Tuberculosis laboratory and treatment registers, with new case notification information, are maintained at township health departments and the Union Tuberculosis Institute where presumptive tuberculosis cases are occasionally referred to from townships health centres for diagnosis. For sake of reference, when discussing data from the Union Tuberculosis Institute and township health departments, they will be collectively referred to as THDs.

**Fig. 1:**
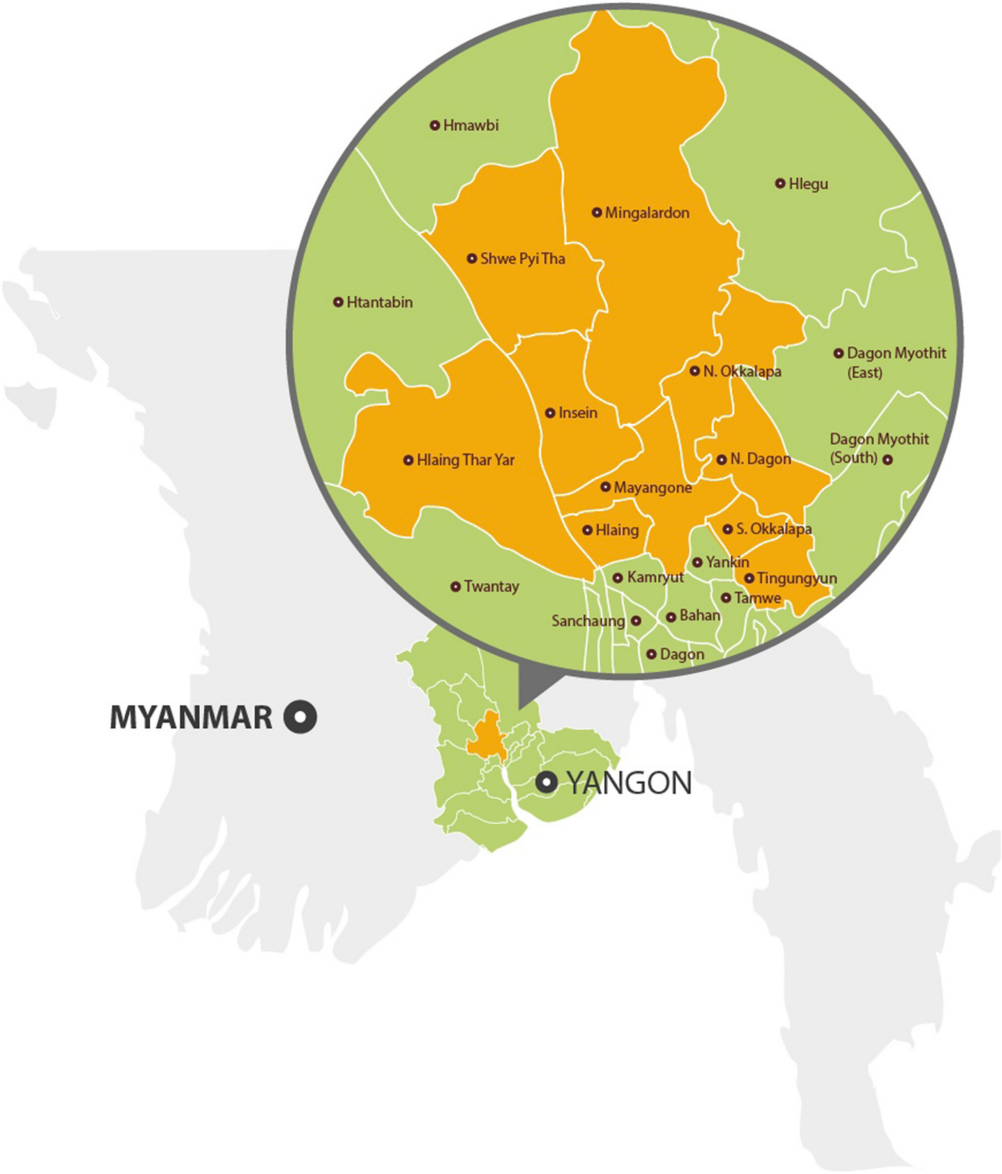
Map of Yangon showing study townships

Our study was conducted in ten townships across Yangon, in which the NTP was receiving programmatic support from the USAID-funded Control and Prevention-Tuberculosis Project, which is led by an international NGO, Family Health International 360 (FHI 360). The townships were: Hlaing, Hlaing Thar Yar, Insein, Mayangone, Mingalardon, North Dagon, North Okkalapa, Shwe Pyi Thar, South Okkalapa and Thingangyun (Fig. 1).

### Participants and data collection

Notification information for newly diagnosed tuberculosis patients is recorded in paper registers (laboratory and treatment registers) at the Union Tuberculosis Institute or the townships health centres. As no electronic records currently exist, and there is no single paper register containing comprehensive information on newly diagnosed tuberculosis patients, we compiled the data from the tuberculosis registers and patient treatment cards to create a single electronic database of all adult tuberculosis patients diagnosed over a six-month period, from October 2014 to March 2015.

We included all patients aged over 18 years with laboratory confirmed smear-positive pulmonary tuberculosis in the study. Our exclusion criteria were: missing information on age or gender; age under 18 years; extra-pulmonary or smear-negative tuberculosis; culture or GeneXpert confirmed drug resistant tuberculosis. Smear-microscopy was conducted at the Union Tuberculosis Institute and township health centre laboratories and bacterial load was graded according to standard guidelines as: scanty, 1+, 2+, or 3 + [30].

Ethical clearance was gained from LSHTM Research Ethics Committee, the FHI 360 Protection of Human Subjects Committee and locally, through the Myanmar Ministry of Health.

### Data analysis

De-identified patient level data was transferred from Excel to Stata version 11 (StataCorp, 2009) for analysis. We conducted a descriptive cross-sectional analysis of sex differences in new tuberculosis diagnoses at the THDs. We calculated the proportion of females among total smear-positive cases diagnosed in different age groups, townships and smear microscopy grading categories, along with the 95 % confidence intervals for each proportion. Proportions were compared using the z-test.

We also analysed published data from the 2009–2010 national tuberculosis prevalence survey, which included 51,367 adults from 70 clusters across the country; after screening through a chest x-ray and face-to-face interview about symptoms, sputum specimens from participant with signs of tuberculosis were tested using sputum microscopy and culture [3]. The prevalence survey identified 123 smear-positive cases and 188 culture-positive cases. We calculated the proportion of females among patients diagnosed using smear microscopy, and among patients using culture when smear microscopy was negative. We then calculated the number of additional cases detected from the use of sputum culture in men and women, and expressed this as a proportion of the cases found through smear microscopy alone. Finally, we made a simple projection of the number of additional cases that would be diagnosed in our study townships if culture was used on all symptomatic patients who are negative on smear microscopy, assuming the same additional case finding yield as occurred in the prevalence survey.

## Results

During the study period, 1390 new smear-positive pulmonary tuberculosis patients were diagnosed at the ten study THDs. Of these, 19 patients were excluded owing to missing information about age and sex. Complete information was available for 1371 patients (99 %), and these were included in the analysis.

Overall, 415 (30 %, 95 % *CI* = 28–33) of patients diagnosed at THDs were females (Table 1). Males outnumbered females in all ten townships; however, there was evidence of variation across townships, with the proportion of female patients ranging from 21 % to 37 % (*p* = 0.0172). There was also strong evidence of variation by month of diagnosis, with female patients comprising 37 % of diagnoses in February 2015 and 23 % in March 2015 (*p* = 0.0004).

**Table 1:** Sex differences in TB notification data from ten townships in Yangon

Variable	Female	Male	Total	Proportion Female (95 % CI)
Patients	415	956	1371	30 % (28–33)
Township				
Hliang	21	50	71	30 % (19–41)
Hliang Tharyar	88	152	240	37 % (31–43)
Insein	66	123	189	35 % (28–42)
Mayangone	21	47	68	31 % (20–42)
Mingaladon	49	119	168	29 % (22–36)
North Dagon	26	79	105	25 % (17–33)
North Okkalapa	63	155	218	29 % (23–35)
Shwe Pyi Thar	33	75	108	31 % (22–40)
South Okkalapa	33	101	134	25 % (18–32)
Thingangyun	15	55	70	21 % (11–31)
Month of diagnosis				
October 2014	71	151	222	32 % (26–38)
November 2014	55	154	209	26 % (20–32)
December 2014	67	129	196	34 % (27–41)
January 2015	69	161	230	30 % (24–36)
February 2015	93	158	251	37 % (31–43)
March 2015	60	203	263	23 % (18–28)
Age group				
18–24	90	93	183	49 % (42–56)
25–34	95	237	332	29 % (24–34)
35–44	77	249	326	24 % (19–29)
45–54	63	199	262	24 % (19–29)
55–64	42	111	153	27 % (20–34)
Above 64	48	67	115	42 % (33–51)
Smear-microscopy grade				
Scanty	36	68	104	35 % (26–44)
1+	129	324	453	28 % (24–32)
2+	85	162	247	34 % (28–40)
3+	162	389	551	29 % (25–33)
Unknown	3	13	16	19 % (0–38)

The analysis of sex differences across different age groups indicated that the proportion of females diagnosed in the youngest and oldest age groups was higher than in middle (25 to 64 years) age groups. The proportion of females among patients aged between 25 and 64 was 26 % as compared to 49 % among patients aged between 18 and 24 (*p* < 0.0001) and 42 % among patients aged over 64 years (*p* < 0.0001). Smear microscopy grading of sputum specimens was not substantially different between male and female smear-positive tuberculosis patients.

Analysis of sex differences in the prevalence survey data, presented in Table 2, indicated that culture detected a substantial number of cases that were not diagnosed using smear microscopy, and that there was a relatively greater increase in case detection in women than men (200 % additional case finding yield in women and 134 % in men). Since the overall number of cases detected in the prevalence survey was small, however, there was only weak evidence to suggest that use of a more sensitive diagnostic tool, culture, resulted in a higher proportion of females diagnosed than use of smear microscopy only (37 % versus 28 %, *p* = 0.11).

**Table 2:** Sex segregated analysis of additional case finding through the use of culture on sputum that was negative on smear microscopy during the prevalence survey and projection of additional case finding at THDs through use of culture

	Prevalence survey (microscopy positive)			Prevalence survey (culture positive when microscopy negative)			Additional cases found using culture		Projected case finding at THDs through use of culture		
Age Group	Female	Male	Proportion Female (95 % CI)	Female	Male	Proportion Female (95 % CI)	Female	Male	Female	Male	Proportion Female (95 % CI)
15–24^a^	2	3	40 % (−3–83)	1	5	17 % (−13–47)	50 %	167 %	135	248	35 % (30–40)
25–34	6	15	29 % (10–48)	14	17	45 % (27–63)	233 %	113 %	317	506	39 % (36–42)
35–44	12	24	33 % (18–48)	14	26	35 % (20–50)	117 %	108 %	167	519	24 % (21–27)
45–54	7	18	28 % (10–46)	11	30	27 % (13–41)	157 %	167 %	162	531	23 % (20–26)
55–64	4	15	21 % (3–39)	11	14	44 % (25–63)	275 %	93 %	158	215	42 % (37–47)
Above 64	4	13	24 % (4–44)	19	26	42 % (28–56)	475 %	200 %	276	201	58 % (54–62)
Total	35	88	28 % (20–36)	70	118	37 % (30–44)	200 %	134 %	1245	2238	36 % (34–38)

^a^Data from the Township Health Centres was only collected from patients aged over 18

## Discussion

Our study of tuberculosis diagnosis in ten townships in Yangon, the first to systematically compile and analyse routine operational data from tuberculosis diagnostic centres in Myanmar, found that the number of women diagnosed at township health centres is much lower than the number of men. Women represented only 30 % of smear-positive cases diagnosed, which is lower than the average of 40 % (all new tuberculosis diagnoses) across South East Asia [11].

While fewer women than men were diagnosed in all townships, there was substantial variation in the proportion of females comprising total diagnoses at individual THDs. This may indicate that gender-related differences in access to THDs or being diagnosed at THD may be operating. The influence of diagnostic centre characteristics, such as opening hours, accessibility on foot, and size of facility has been found in other settings to influence relative numbers of men and women with tuberculosis symptoms using the centres [31]. Healthcare quality - specifically health worker adherence to testing protocols for patients reporting tuberculosis-related symptoms, and any biases in referring of patients for sputum testing - could influence sex differences in diagnosis at individual THDs. At least three studies have now found that women who present with the same symptoms as men at health centres are less likely to be tested for tuberculosis and these studies speculate that health workers may hold strong stereotypical views about the ‘typical’ tuberculosis patient being male rather than female [9, 28, 32].

We identified a sharp decrease in the proportion of females among total diagnoses in the 25 to 64 age groups, as compared to the 18 to 24 and over 65 age groups. A study in Bangladesh found a similar decrease in the relative number of diagnoses from women aged 18 to 25 as compared to women aged 25 to 64, but did not find that the oldest (over 65) age group had a relatively higher number of diagnoses from women, as we did. In contrast to our findings, other studies have found that older age is associated with greater barriers to diagnosis in women [21, 26]. Overall, evidence about interplays between age and gender dynamics in tuberculosis healthcare seeking and diagnosis is limited, with differences in stigma, healthcare seeking behaviour and household responsibilities in diverse contexts likely to play an important role. Some studies have found that tuberculosis stigma has greater socio-economic consequences for women, and particularly affects care-seeking decisions of young women who are close to marriageable age or recently married [33, 34]. There are few studies exploring stigma and barriers to accessing tuberculosis care in Myanmar. We found only one study focusing on male factory workers in Yangon, which found that stigma and fears related to tuberculosis were common [35]. Data from neighbouring Bangladesh indicates that female tuberculosis patients delay seeking care and self-medicate more often than men [36]; barriers influencing women’s decisions to visit health facilities include the need for a male to accompany them, time constraints and responsibilities for household chores [33, 36, 37].

Another factor that may be contributing to higher notifications from women aged 18 to 24 is potentially higher HIV prevalence amongst this group. Age and gender patterns of tuberculosis are influenced by the HIV epidemic in other countries, such as South Africa [38]. Overall 8.5 % of new tuberculosis patients are HIV sero-positive in Myanmar, but information about age and sex differences in TB-HIV co-infection is not available [39]. Studies of female sex workers in Myanmar have indicated higher HIV prevalence in younger women [40]; HIV-TB co-infection in Myanmar, and its impact on the tuberculosis epidemic, requires further investigation.

Reasons for the observed sex differences by month of diagnosis are not clear. Since the study was conducted in an urban setting, Yangon, we are not aware of any agricultural activities that could influence healthcare seeking behaviour. There is a marked rainy season between May and October which may, we can hypothesise, impact upon health centre functioning or access to services. Analysis of operational data from other countries identified similar variations across the year, and the authors speculated changes in the weather, school holidays or national holidays affecting healthcare opening may play a role [41].

We explored whether there was evidence of female tuberculosis patients in Yangon having a lower bacterial load in their sputum; this has been found in other settings, and offered as an explanation for lower case finding in women when smear microscopy is used as the primary diagnostic tool [42, 43]. We found no sex difference in the bacterial load of sputum submitted for testing at our study THDs [43, 44]. However, our analysis of sex differences in the prevalence survey data did indicate that smear microscopy may be missing a relatively higher proportion of cases in women than men, as indicated by the (sex segregated) additional case finding by using culture on smear-negative sputum.

Our simple projection of additional case finding at the THDs from the use of culture on sputum from symptomatic patients testing negative on sputum smear microscopy, extrapolated from our analysis of the prevalence survey data, indicated that 1245 (200 %) more cases would be detected in women and 2238 (134 %) in men. This would result in women representing 36 % of smear-positive cases diagnosed instead of 30 %.

While use of culture or other more sensitive diagnostic tools such as Xpert MTB/RIF instead of smear microscopy could lead to a substantial increase in tuberculosis diagnoses [45], the cost and infrastructure requirement makes widespread use very challenging, and evidence on the operational effectiveness and cost-effectiveness in low-resource setting is limited. Owing to the high prevalence of MDR-TB in Yangon, the NTP has started testing all presumptive tuberculosis patients in Yangon using Xpert MTB/RIF. Analysis of operational data from the wider use of a more sensitive diagnostic tool, the Xpert MTB/RIF test, will be useful in demonstrating whether there is a sex difference in case finding when sensitivity of the diagnostic tool is altered. Our initial evidence from Myanmar, which has a particularly low case detection of tuberculosis in women, indicates that more sensitive diagnostic tools may benefit female tuberculosis patients differentially. There is already strong evidence that improving the quality of instructions provided to patients at health facilities can have a differential impact on improving case finding in women [27]. Further operational studies to assess the sex specific incremental case finding yield and cost-effectiveness of alternative diagnostic algorithms are needed to inform programmatic strategy decisions. These studies will be particularly useful in settings such as Myanmar where there is markedly low case detection in women, and substantial funding being allocated to improve tuberculosis control.

The key strength of our study is that we had a well-trained team to collect data comprehensively from all available paper records in a number of locations in order to produce an electronic database on new tuberculosis diagnoses in our ten study townships. Since maintenance of paper registers was erratic, this data compilation was a substantial undertaking. A limitation of the study is that we did not have the resources available to explore and collate data on the number of males and females attending THDs with respiratory complaints or the number referred for sputum testing; it is not clear whether this data would be available consistently in THDs for collation. Such data would allow a useful investigation of barriers to diagnosis of men and women at different stages of the diagnostic process, and has resulted in important insights about potential missed cases in women in other settings [28, 46].

It is also important to consider that our study, and most other studies on sex differences in tuberculosis diagnoses, only includes data from public sector health facilities. In Myanmar, it is estimated that over 70 % of tuberculosis patients first seek care at private healthcare providers; to understand sex differences fully it is essential to investigate tuberculosis diagnosis and management in the private sector [47].

## Conclusions

This paper presents the first analysis of sex differences in tuberculosis diagnoses in Myanmar, where only 34 % of nationally reported cases are from women. Our initial evidence suggests that socio-demographic and health service-related factors may be influencing sex differences in tuberculosis notifications. Specifically, our analysis indicates that low sensitivity of tuberculosis diagnosis at health centres may lead to a potential under-diagnosis of tuberculosis among women. Improving the sensitivity of tuberculosis diagnosis at health centres could therefore be investigated as a programmatic area that may reduce sex differences in tuberculosis diagnoses in Myanmar.
